# Housing and Sanitation in Dundee

**Published:** 1905-09-30

**Authors:** 


### Housing and Sanitation in Dundee.

The publication of the report on the feeding of school children in Blackburn, to which we directed attention last week, has speedily been followed by a report of a more extensive character, describing the housing and general social conditions of the industrial classes of Dundee. This report is the outcome of work undertaken by a voluntary local organisation, the Dundee Social Union, which for the last seventeen years has been engaged in an endeavour to improve the housing accommodation of the city, and which, in the words of the report, has felt that the time was come for ascertaining, "by direct and careful examination, under what conditions the life of a not inconsiderable portion of the community is carried on." Under the advice of the Right Hon. C. Booth, Mr. Rowntree, and others, it was determined that the detailed work of the inquiry should be put into experienced hands, and that what might be called amateur assistance should not be employed. The direction and oversight of the whole were entrusted to two ladies—Miss M. L. Walker, who has acted for many years as honorary superintendent of the Dundee Social Union, and Miss Mona Wilson. A staff of trained assistants aided in the work. The advice and active help of the Medical Officer of Health, Dr. Templeman, were freely given, and five of the city doctors undertook special investigations. The results of their medical inspections of school children were published last February, in advance of the general report, and attracted much attention at the time.

Dundee has a population of over 160,000 mainly dependent upon a declining industry—that of jute manufacture—which is chiefly in the hands of badly paid female workers. The natural consequences are a general inability to pay for decent dwellings, a low standard of living, an enormous infantile mortality, and a very poor condition of health and development among the children who survive. In addition to these evils, and notwithstanding the evidence afforded by the very existence of the Social Union of a desire for better things on the part of prominent citizens, it is difficult to resist the conclusion, on perusal of the report as a whole, that there must have been a considerable tendency to "folding of the hands in sleep" on the part of the city authorities. Thus, we are told that "with bad ventilation, insanitary closets, and filthy ashpits, the stench in congested centres is described by all of the inspectors as sometimes appalling. Tenants introduced to such surroundings, however well intentioned in the matter of decency and personal cleanliness, must in the nature of things lose in time self-respect, and cease to make any effort after better conditions." It is then pointed out that even more frequent emptying of the ashpits would alone be an important reform, and this is clearly a matter which the municipality should undertake.

It is difficult to believe, however, that any substantial improvement in the conditions described by the report will be possible so long as the principal, or almost the only, local industry continues to be in the state which the report describes. It is impossible for a population mainly supported by its women, paid at the ordinary rates of female wages, to be in a cleanly, a healthy, or a prosperous condition; and the sooner this is realised in Dundee, and the sooner the energies of its leading inhabitants are directed towards the introduction of some new form of industry, the sooner they will be relieved from what the report calls "certain social evils, which may be described fittingly and without exaggeration as a disgrace and national peril." We all know of examples, that of Coventry, for instance, in which the decline of an original manufacture and a period of distress consequent thereupon, have been followed by the adoption of another and by a return of prosperity, and nothing less than some change of this description can restore Dundee to her rightful position. At present, according to the report, "without women's labour the city would sink to the level of a small burgh; as a manufacturing centre it would possibly cease to exist." With women's labour as the chief support of its single local industry, it has the conditions of housing partially described above, it has an infantile mortality of 176 per 1,000 births (against 151 in Aberdeen, 146 in Glasgow, 136 in Paisley, and 130 in Edinburgh), and it has a population of children falling very considerably below the accepted standards in height, weight, and chest measurement. A small inquiry quoted in the report shows that 240 mothers who had been working both before and after marriage had given birth to 885 children, of whom 520 were dead; 91 mothers who had either worked only before marriage or not at all had given birth to 460 children, of whom 195 were dead. There were 630 living children and 715 dead for the 331 families, and all of the dead children, with seven exceptions, lived less than five years, and 630 of them less than one year. No special inquiry into the question of the under-feeding of children is recorded, but the medical sections of the report bristle with evidences of imperfect nutrition and of its consequences, and it is manifest that the survivors of the infantile mortality which has been described are not calculated to grow up into healthy or capable men and women. As viewed through the medium of the report, Dundee can only be regarded as a breeding-ground for degenerates upon a scale probably unapproached elsewhere; and the conditions described are so grave that they cannot be treated as matters merely of local concern. They concern the honour and the well-being of the country in which they are suffered to exist, and they imperatively demand the attention, not only of the leading inhabitants of Dundee itself, but also of the Scottish members of the Legislature and of the Scottish captains of industry. If some new form of trade or manufacture could be established in Dundee the city might within a reasonable time be expected to emerge from the condition in which it is now said to exist; and, if this be impossible, it would be better for it to be forsaken by its population than to retain them in a condition fitly described as one which is a national disgrace.

